# Prolactin in combination with interferon-β reduces disease severity in an animal model of multiple sclerosis

**DOI:** 10.1186/s12974-015-0278-8

**Published:** 2015-03-19

**Authors:** Simon Zhornitsky, Trina A Johnson, Luanne M Metz, Samuel Weiss, V Wee Yong

**Affiliations:** Hotchkiss Brain Institute and the Department of Clinical Neurosciences, Cumming School of Medicine, University of Calgary, 3330 Hospital Drive NW, Calgary, AB T2N 4N1 Canada

**Keywords:** Prolactin, Multiple sclerosis, Interferon-β, Experimental autoimmune encephalomyelitis

## Abstract

Previous work has demonstrated that the hormone prolactin promotes oligodendrocyte precursor proliferation and remyelination following lysolecithin-induced demyelination of the mouse spinal cord. Prolactin, however, can elicit pro-inflammatory responses, and its use in the prototypical demyelinating and inflammatory condition, multiple sclerosis (MS), should thus be approached cautiously. Here, we sought to determine whether recombinant prolactin could alter the course of experimental autoimmune encephalomyelitis (EAE), an inflammatory animal model of MS. Consistent with previous literature, we found that prolactin activated leukocytes *in vitro*. Daily treatment with prolactin from around the time of onset of clinical signs, for 9 (days 9 to 17) or 25 (days 9 to 33) days did not increase clinical or histological signs of EAE over that of vehicle-treated mice. Instead, the combination of prolactin and a suboptimal dose of recombinant murine interferon-β resulted in (days 9 to 17 group) or trended towards (days 9 to 33 group), a greater amelioration of clinical signs of EAE, compared to either treatment alone or to vehicle controls. Histological analyses corroborated the clinical EAE data. These results suggest that prolactin may be beneficial when administered in combination with interferon-β in MS.

## Introduction

Multiple sclerosis (MS) is a chronic T and B cell-mediated inflammatory disorder of the central nervous system (CNS) associated with progressive oligodendrocyte and neuronal loss, axonal degeneration, and demyelination [1]. Prolactin is a pituitary hormone that stimulates milk production in mammals, and it may be implicated in the pathophysiology of MS [2,3]. Interestingly, MS tends to undergo remission during mid-late pregnancy, where prolactin levels are known to be at their peak during the third trimester of pregnancy [4-7]. Breastfeeding promotes further prolactin release, even though levels would otherwise fall post-partum [8,9]. Importantly, a recent meta-analysis (12 studies; *n* = 1,558) found that women with MS who breastfed were almost half as likely to experience a post-partum relapse compared to women who did not [10]. The data are even more compelling when considering only studies that examined exclusive breastfeeding [11,12].

In animals, our group showed that pregnant mice have an enhanced ability to remyelinate lysolecithin-induced white matter lesions [13]. This was observed via an increase in proliferation of oligodendrocyte precursor cells, oligodendrocyte generation, expression of myelin basic protein, and in the number of myelinated axons - effects that were stimulated by prolactin infusion. Others have revealed that prolactin may possess pro-inflammatory properties. For example, there is evidence that prolactin promotes the activation of B cells cultured from the blood of MS patients [14]. Additionally, T cell cultures treated with prolactin have an elevated T helper 1 pro-inflammatory profile and decreased suppressive function of regulatory T cells [15]. Preclinical studies suggest that reduction of prolactin levels via treatment with dopamine D_2_ agonists (bromocriptine and dihydroergocryptine) reduces severity of experimental autoimmune encephalomyelitis (EAE), an animal model of MS [16-19]. On the other hand, a recent study revealed that prolactin receptor- and prolactin-knockout mice develop a delayed onset EAE, compared with littermate control mice, but with full clinical severity [20]. Importantly, no study of the direct administration of purified prolactin in EAE has been conducted to date.

The present study examined the effect of purified recombinant prolactin in EAE at different periods surrounding the onset of neurological signs and with/without concomitant interferon-β (IFN-β), a standard disease modifying therapy in MS. The addition of IFN-β was explored because of the potential pro-inflammatory properties of prolactin, making it prudent to combine its use with an immunomodulator commonly used in MS.

## Methods

Recombinant murine prolactin was tested in virgin C57BL/6 female mice aged 8 to 10 weeks. EAE was induced at day 0 by 50 μg myelin oligodendrocyte glycoprotein (MOG), peptide 35-55, emulsified in Complete Freund’s Adjuvant supplemented with 4 mg/kg of mycobacterium, as described elsewhere [21]. Mice were also administered two intraperitoneal injections of pertussis toxin (List Biological Labs, Hornby, ON, USA), on days 0 and 2. Intraperitoneal injections of 20 μg/mouse of prolactin (Harbor-UCLA, Torrance, CA, USA) were applied every morning, either for 9 (days 9 to 17) or 25 (days 9 to 33) consecutive days. Previously, we found that the same dosing regimen promoted myelin repair in virgin female mice of a similar age. In the 9-day experiment, while mice were treated from days 9 to 17, they were sacrificed on day 21. In the 25-day experiment, mice were sacrificed on the day of the last treatment itself. Day 9 was the time period when onset of clinical signs of EAE was anticipated. Groups of mice also received suboptimal doses (20,000 IU/mouse) of recombinant murine IFN-β (PBL Biomedical Laboratories, Piscataway, NJ, USA) administered subcutaneously once every 2 days, alone or in combination with prolactin. Vehicle was phosphate-buffered saline.

EAE clinical disease scores were obtained by using a 15-point scale that totals the degree of disability of the tail (scored from 0 to 2) and all four limbs (each limb scored from 0 to 3) [22]. When the sum of scores (burden of disease) was tabulated, this represents the daily clinical score per mouse, added over the course of the experiment for that mouse.

Histological analyses were performed on spinal cord specimens retrieved at the end of treatment. The degree of inflammation and demyelination of the spinal cord was assessed through hematoxylin and eosin and luxol fast blue (H&E/LFB), as previously described [21]. Animal studies were performed according to ethical policies outlined by the Canadian Council for Animal Care and the University of Calgary.

### Antigen-recall assay

Mice were sacrificed, and lymph nodes were removed on day 10 after MOG immunization. Lymph nodes (LNs) were dissociated, and single cell suspensions were collected in phosphate-buffered saline. LN cells were plated in round-bottom plates at a density of 250,000 cells/100 μl in RPMI containing 1% mouse serum (Invitrogen, Carlsbad, CA, USA). The LN populations served to provide T cells for subsequent antigen-recall proliferation. Antigen-presenting cells (APCs) were harvested from spleens taken from non-immunized animals. Spleens were dissociated and subjected to red blood cell lysis. Cells were re-suspended in RPMI containing 1% mouse serum. APCs were irradiated at room temperature with a Gamma Cell 1000 (Nordion International Inc., Vancouver, Canada) using Cs-137 for 14 min at 3,000 rad, which was then followed by incubation of the APC cells with 20 μg/ml MOG 35-55 for 30 min at 4°C. The APCs (250,000 APCs/100 μl) were then added to the LN cells described above for a total of 500,000 cells/well. Recombinant mouse prolactin was added (1, 10, and 30 nM) and incubated for 3 days. The doses were chosen from previous literature that had suggested a pro-inflammatory role for prolactin *in vitro* on T lymphocytes. 3H-thymidine was added 18 h before the harvesting and collection of cells for measurement of thymidine uptake. Cells were pulsed with 1 μCi of [H3] thymidine (Perkin Elmer, Waltham, MA, USA) to determine the proliferative state of the cultures. After incubation, cells were harvested using a PHD cell harvester (Brandel Inc., Gaithersburg, MD, USA) and [H3] thymidine incorporation was determined by using a Beckman LS3801 scintillation counter (Beckman Coulter, Mississauga, Canada).

### Statistics

Statistical analysis was performed using SPSS Statistics v.22.0 (IBM Corporation, Armonk, NY, USA, 2013). Statistical differences between groups were evaluated using a non-parametric Kruskal-Wallis analysis. Multiple comparisons were performed using the Mann-Whitney *U* test. In all tests, *P* ≤ 0.05 was considered statistically significant.

## Results

### Prolactin promotes MOG antigen-specific proliferative responses

Lymphocytes harvested from the lymph nodes of mice sacrificed at day 10 post-MOG immunizations revealed increased lymphocyte recall responses to MOG antigen (data not shown). Prolactin significantly increased the overall proliferative index of cells responding to a second exposure to MOG peptide. The addition of prolactin increased thymidine uptake by splenocytes in a dose-dependent manner with the maximal response obtained with 30 nM prolactin (data not shown).

### Prolactin in combination with IFN-β reduces EAE severity: days 9 to 17 with sacrifice at day 21

**Figure 1:**
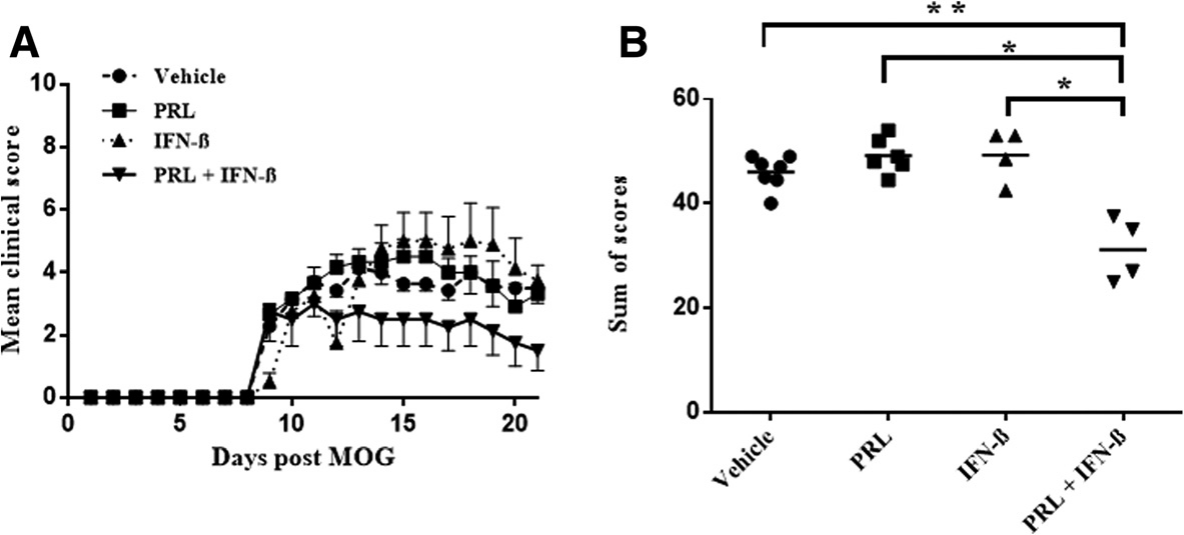
**The combination of prolactin (PRL) and IFN-β attenuated EAE clinical severity, relative to vehicle and either treatment alone. (A)** Mean clinical EAE scores from Tx days 9 to 17 with sacrifice at day 21 (bars indicate SEM). **(B)** Sum of EAE scores from Tx days 9 to 17 with sacrifice at day 21, with each circle, square, or triangle depicting a separate animal: **P < 0.01; *P < 0.05. There were seven vehicle mice, six PRL-alone animals, and four each of IFN-β alone or the combination group.

The pro-remyelinating effects of prolactin suggest its potential use in demyelinating conditions including MS [13]. However, given the potential of prolactin to promote pro-inflammatory responses in T and B lymphocytes [14,15], it is important to consider that prolactin may increase disease severity of MS or its model, EAE. Thus, we treated EAE-afflicted mice with recombinant prolactin from their onset of clinical signs, daily for 9 days (days 9 to 17), with animals killed at day 21 for histology. The dose of 20 μg prolactin/mouse/day was chosen as this was the pro-remyelinating dose in the lysolecithin demyelinating model in mice [13]. We found that EAE-afflicted mice treated with vehicle attained peak clinical severity of between 4 and 6 on the 15-point scale [22], representing tail and hind-limb disability (Figure 1A); this modest disease severity provided room to reveal a pro-disease exacerbating effect, as EAE-afflicted mice often have involvement of forelimb disability in other experiments (data not shown). Prolactin treatment did not exacerbate EAE clinical severity over that of vehicle-treated EAE mice (Figure 1A), and a suboptimal dose of recombinant murine IFN-β also did not affect EAE clinical severity. Interestingly, the combination of prolactin and IFN-β resulted in a reduction of sum of daily clinical scores (Figure 1A) to an average of 2 on the 15-point scale, representing tail disability but without hind-limb involvement.

To better document the disease-lowering effect of prolactin, we added the daily clinical score over the course of the 21 days on a per mouse basis; this sum of scores reflects the burden of clinical disease for that mouse over the course of the experiment. The sum of EAE scores was significantly different between the four groups (*χ*^2^ = 10.941; *P* = 0.012; Figure 1B), and *post hoc* analyses demonstrate that the combination of prolactin and IFN-β resulted in significantly lower sum of scores, relative to prolactin-alone (*Z* = −2.558; *P* = 0.011), IFN-β-alone (*Z* = −2.323; *P* = 0.020), and vehicle (*Z* = −2.652; *P* = 0.008) (Figure 1B). The difference among the four groups was still significant when analyzing sum of scores from days 9 to 17 only (*χ*^2^ = 12.01; *P* = 0.007).

**Figure 2:**
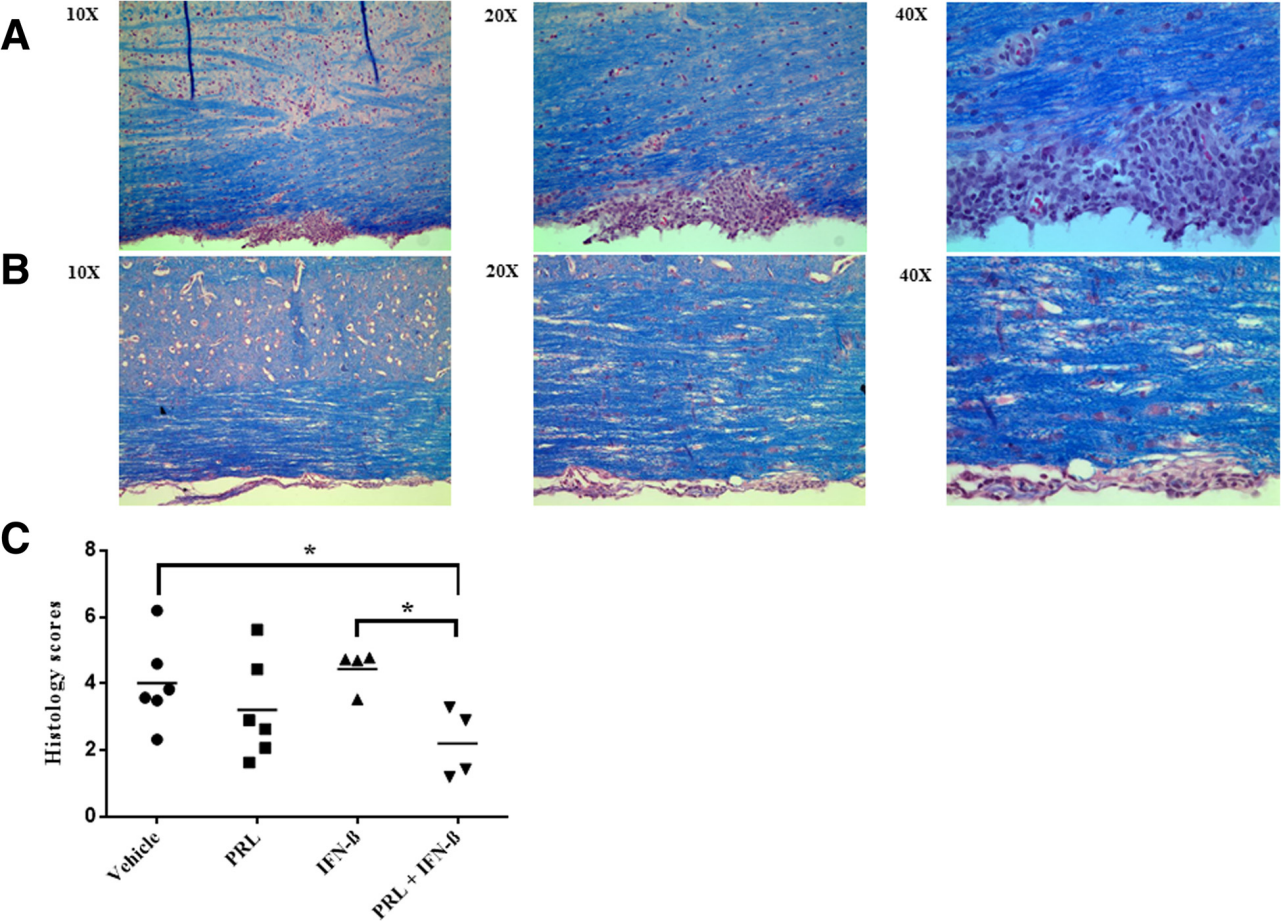
**The combination of PRL and IFN-β reduced histopathology in the spinal cord.** Histology at day 21 for a representative vehicle mouse (histology score = 4) **(A)** or from a representative mouse in the combination group **(B)** (histology score = 2). The × 10, ×20, and × 40 depict the original magnification as captured by the respective objective lens. **(C)** Mean histology scores from day 21, where each display is of a separate mouse: *P < 0.05.

Animals were sacrificed at day 21, and H&E/LFB histological analyses encompassing general histology, demyelination, and cellular infiltrates were conducted (Figure 2). There was a borderline significant difference in histological scores (*χ*^2^ = 7.594; *P* = 0.055). *Post hoc* analysis revealed that the combination of prolactin and IFN-β resulted in significantly lower histological scores, compared to vehicle (*Z* = −2.132; *P* = 0.033) and IFN-β-alone (*Z* = −2.309; *P* = 0.021). There was no significant difference between the combination of prolactin and IFN-β *versus* prolactin-alone in terms of histological scores.

### Extended treatment with prolactin (days 9 to 33) did not promote EAE disease severity

**Figure 3:**
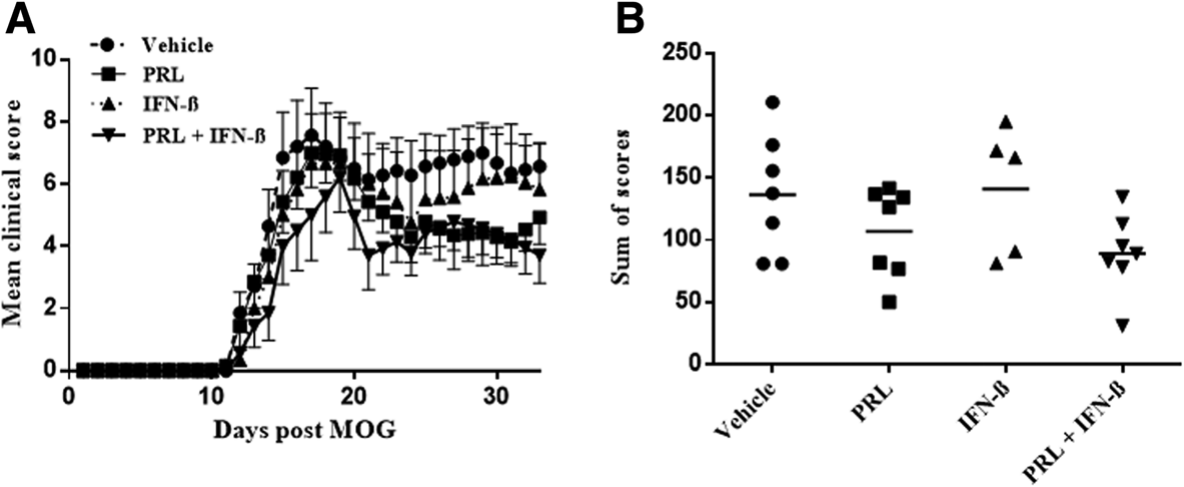
**A trend towards reduction of clinical scores was observed in EAE mice on long-term prolactin or combination treatment. (A)** Mean clinical EAE scores in animals treated from Tx days 9 to 33 (bars indicate SEM). **(B)** Sum of EAE scores from Tx days 9 to 33. Statistical significance was not found although there was a tendency for the prolactin-alone, or combination treatment group to have lower clinical disease scores. There were seven mice each in the vehicle, prolactin-alone, or combination group, while there were five animals in the IFN-β-alone group.

Given that the use of prolactin may be for an extended period in chronic conditions such as MS, we sought to address a longer treatment period of EAE-afflicted mice with prolactin. From day 9, daily treatment with prolactin, and every other day of IFN-β, was effected until day 33. In this experiment, the EAE-afflicted mice treated with vehicle had a higher disease score, compared to the days 9 to 21 experiment, of between 6 and 8 on the 15-point scale; this represents disability occurring in the tail and hind limbs and also involvement of the forelimbs. Perhaps because of this higher severity, prolactin-alone, or prolactin plus IFN-β, did not result in a significant reduction in clinical score compared to vehicle-treated mice, although a tendency towards reduction was observed (*χ*^2^ = 4.830; *P* = 0.185; Figure 3A,B). Importantly, prolactin did not enhance disease severity.

**Figure 4:**
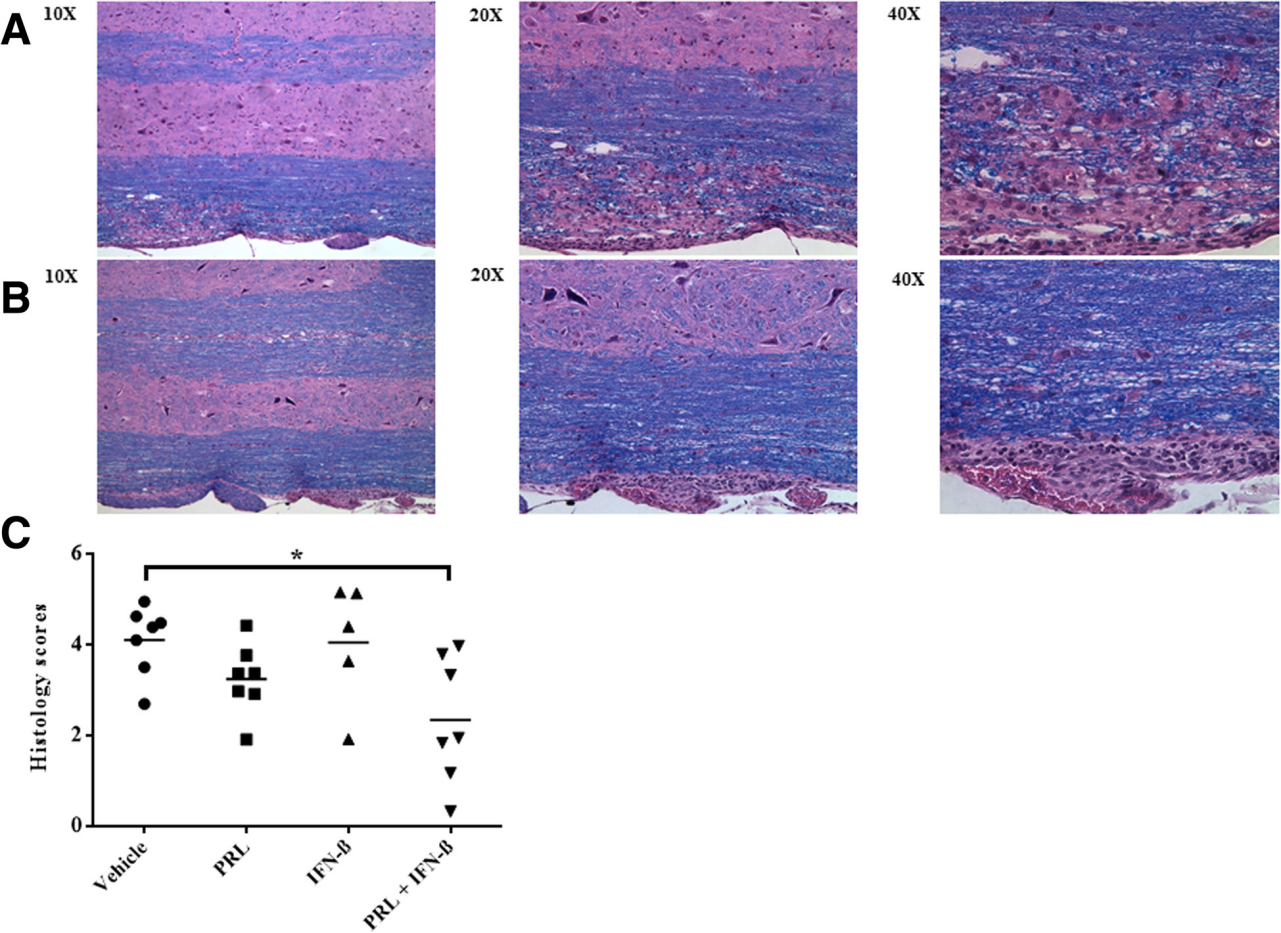
**The combination of PRL + IFN-β attenuated histology scores in the spinal cord. (A)** Histology from day 33, depicting inflammation and demyelination, in a representative vehicle mouse (histology score = 4), or from a representative animal in the combination group **(B)** (score = 2). **(C)** Mean histology scores from day 33, where each circle, square, or triangle is of a separate mouse. *P < 0.05.

For histological analyses, there was a significant difference detected (Figure 4; *χ*^2^ = 8.477; *P* = 0.037). Multiple comparisons revealed that the combination of prolactin plus IFN-β resulted in significantly lower histology scores, relative to vehicle (*Z* = −2.492; *P* = 0.013), and there was a trend towards reduced histology scores, compared to IFN-β-alone (*Z* = −1.868; *P* = 0.062). There was no significant difference between the combination of prolactin and IFN-β and prolactin-alone in terms of histology scores.

## Discussion

Previous work has demonstrated that the hormone prolactin promotes the proliferation of oligodendrocyte precursor cells and remyelination in the lysolecithin model of demyelination in mice [13]. That study also showed that white matter changes occurred in the maternal CNS, and these changes were initiated during early pregnancy and include increases in proliferation of oligodendrocyte precursor cells, oligodendrocyte generation, expression of myelin basic protein, and, ultimately, an increase in the number of myelinated axons. In addition, we found that prolactin signaling was necessary and sufficient for the regenerative effects of pregnancy and that prolactin treatment of virgin mice enhanced their remyelinating capacity following demyelination [13]. Pro-regenerative effects were also observed by Möderscheim *et al*. [23] wherein prolactin exerted trophic and pro-proliferative effects on glia.

The pro-remyelinating capacity of prolactin suggests its utility in demyelinating conditions such as MS, except that this must be weighed against reports that prolactin can have pro-inflammatory roles. A pro-inflammatory role for prolactin has been reported for T lymphocytes previously by stimulating splenocytes *in vitro* using concanavalin A [17,18]. These cells displayed lymphoproliferation in a dose-dependent manner that could be antagonized with the use of corticosteroids with lymphoproliferation observed at concentrations of 5 nM but not 1 nM prolactin in culture [24]. Additionally, the proliferation of splenocytes and thymocytes stimulated with anti-CD3 was further promoted in the presence of 10 nM ovine prolactin as assessed by thymidine uptake [25]. In both cases, the antigen non-specific response was measured and these effects were mimicked by the addition of growth hormone suggesting that prolactin may act similarly to this hormone as a mitogen for cell proliferation. It remains to be shown whether prolactin plays a role in stimulating memory or recall responses. Here, the mitogenic effect of prolactin seen previously with anti-CD3 and concanavalin A was replicated in a MOG peptide-specific recall assay, suggesting that prolactin may be pro-proliferative when present during antigen-recall in an ongoing immune response.

A dopaminergic pathway in the hypothalamus-pituitary axis controls the production of prolactin. Treatment with D_2_ agonists lowers prolactin levels, and a number of studies have reported beneficial effects of prolactin suppression in EAE. In one study, bromocriptine given 1 week before immunization significantly decreased serum prolactin levels, and this was accompanied by an inhibition of disease progression in acute EAE [16]. In that study, immunocompetence of bromocriptine-treated animals was restored by additional treatment with either prolactin or growth hormone. A similar study by Riskind *et al*. [17] revealed that induction of acute EAE resulted in a threefold rise in prolactin levels on day 4 after immunization and maintained elevated levels on day 10 before the onset of neurological signs. Bromocriptine significantly reduced the rise in prolactin levels and inhibited disease progression when initiated 1 week after immunization and also in late disease. Another report administered bromocriptine after the onset of clinical signs in acute as well as in chronic relapsing EAE [19]. Their results revealed that bromocriptine suppressed prolactin levels and reduced the severity and duration of clinical signs in acute EAE and the duration of the second attack in chronic EAE. Finally, there is evidence that dihydroergocryptine induced a large reduction of prolactin levels accompanied by a significant improvement in neurological signs of acute EAE when given 2 days before immunization [18]. Taken together, these studies suggest that reduction of prolactin levels by selective D_2_ agonists is effective at reducing disease severity in acute and chronic EAE, supporting a pro-inflammatory effect of prolactin. However, a small clinical trial (*n* = 18) did not find a benefit of bromocriptine in RRMS and progressive MS patients [26]. More recent literature suggests that dopamine may be directly linked to immunomodulation - for example, by inhibiting activated T cell function, modulating Tregs, and altering B cell function [27]. Thus, suppression of prolactin in the aforementioned studies may not be the primary mechanism via which dopamine agonists reduce EAE severity. Taken together, it is currently not clear whether suppression of physiologic levels of prolactin via therapy with D_2_ agonists would benefit patients with MS.

A recent study revealed that prolactin receptor- and prolactin-knockout mice develop a delayed onset EAE, compared with littermate control mice, but with full clinical severity [20]. Because prolactin receptor knockouts have been shown to be hyperprolactinemic, these data suggest that neither high nor low prolactin levels significantly impact EAE. The data are correspondent with previous results showing that prolactin receptor-knockout mice develop normal immune function [28,29].

In view of the uncertainty of the impact of prolactin in MS, we have reviewed the literature and concluded that there is no compelling argument against the use of prolactin in MS [3]. In support, post-partum data of breast-feeding where prolactin levels are expected to be high have indicated that there may be a protective effect of breast-feeding against MS relapses [10-12].

Nonetheless, the use of prolactin in MS must be approached cautiously, and it would be prudent to combine its use with an immunomodulator commonly used in the condition. In the current study, we have used purified recombinant prolactin in EAE. Our data shows that recombinant prolactin-alone had no effect on EAE clinical scores during treatment for 9 or 25 days. Indeed, prolactin did not exacerbate EAE - as would be predicted based on its pro-inflammatory properties - but may have exerted further beneficial properties not apparent in the overwhelming inflammatory and potentially pro-proliferative mechanisms of EAE. With this in mind, we examined the effect of prolactin in the presence of suboptimal doses of the therapeutic immunomodulator IFN-β. Our data suggests that the mixture of these two therapies given over a protracted period after disease onset provides a benefit not observed with either of the single treatment therapies. In addition, observation of spinal cord histopathology of these mice suggests that combination therapy decreased the overall disease burden as reflected by inflammatory infiltrates. Consequently, it is possible that IFN-β counteracted a pro-inflammatory effect of prolactin, thus allowing for the putative remyelinating properties of prolactin to take effect - as previously shown by our group in the lysolecithin model [13].

We were unable to determine whether the improvement in clinical scores in response to combined prolactin and IFN-β treatment could be attributed to remyelination. The EAE model is notoriously difficult to evaluate remyelination, as lesions appear at unpredictable locations, and it is therefore not trivial to address the evolution of demyelination and its subsequent repair. Thus, while the histological analyses (Figures 2 and 4) show reduced inflammation and demyelination in response to combined prolactin and IFN-β treatment, to what extent remyelination has occurred remains unclear.

In conclusion, we sought to determine whether prolactin could alter the course of EAE, either when used alone, or in combination with IFN-β. We found that the combination of prolactin and IFN-β during the 9-day treatment period resulted in a greater amelioration of clinical signs and histological scores of EAE compared to either treatment alone. The combination therapy also resulted in significantly less inflammatory infiltrates with prolonged 9- to 33-day treatment. Consequently, in light of the seemingly beneficial effects of breast-feeding on MS symptomatology, and our promising results in the lysolecithin and EAE models, future trials of prolactin in MS may be warranted. Either prolactin itself, or medications that elevate prolactin levels, may be considered.

## Abbreviations

APCs: Antigen-presenting cells
CNS: Central nervous system
EAE: Experimental autoimmune encephalomyelitis
H&E/LFB: Hematoxylin and eosin and luxol fast blue
IFN-β: Interferon-beta
LNs: Lymph nodes
MS: Multiple sclerosis
MOG: Myelin oligodendrocyte glycoprotein
